# Stiff matrix promotes lung cancer cell migration through down-regulating the Piezo1 channel expression to facilitate Ca^2+^-dependent filopodia formation

**DOI:** 10.1016/j.mtbio.2026.102786

**Published:** 2026-01-12

**Authors:** Xiaoling Jia, Lin Zhao, Juncheng Bai, Lu Wen, Qianyu Meng, Haikun Wang, Junqi Men, Hui Shao, Yingying Guo, Xinlan Chen, Xing Chen, Lin-Hua Jiang, Yubo Fan, Huawei Liu

**Affiliations:** a Key Laboratory for Biomechanics and Mechanobiology of Ministry of Education, Beijing Advanced Innovation Center for Biomedical Engineering, School of Biological Science and Medical Engineering, and with the School of Engineering Medicine, Beihang University, Beijing, China; b Sino-UK Joint Laboratory of Brain Function and Injury of Henan Province, and Department of Physiology and Pathophysiology, Xinxiang Medical University, Xinxiang, China; c Henan Key Laboratory of Neurorestoratology and Protein Modification, The First Affiliated Hospital, Henan Medical University, Xinxiang, China; d School of Biomedical Sciences, Faculty of Biological Sciences, University of Leeds, Leeds, UK; e Department of Stomatology, Medical School of Chinese PLA, The First Medical Center, Chinese PLA General Hospital, No.28 Fuxing Road, Haidian District, Beijing, 100853, China

**Keywords:** Stiff matrix, Piezo1 channel, Lung cancer cell migration, Cofilin, Filopodia formation

## Abstract

Matrix stiffening profoundly influences cancer cell functions and cancer progression, and the mechanosensitive Piezo1 channel is implicated in these processes. Different from what is observed in most solid tumors, the Piezo1 channel in lung cancer is down-regulated and negatively regulates cancer cell migration, but the underlying mechanism is still unclear. Herein, we investigated the role of Piezo1 channel in matrix stiffness regulation of lung cancer cell migration and the mechanisms in A549 cells growing on polyacrylamide (PA) hydrogels with different stiffness. Compared with soft substrate, stiff substrate promoted cell migration, down-regulated Piezo1 expression, favored filopodia formation, as well as restraining the rise in intracellular calcium concentration ([Ca^2+^]_i_). Additionally, blockade or knockdown of Piezo1 channel promoted, whereas its activation suppressed, cell migration and filopodia formation. Furthermore, reducing the [Ca^2+^]_i_ promoted cell migration and filopodia formation. Finally, stiff substrate induced cofilin phosphorylation, which was enhanced by inhibiting the Piezo1 channel or reducing the [Ca^2+^]_i_ and, conversely, suppressed by activating the Piezo1 channel. Collectively, our study has revealed that stiff matrix down-regulates the Piezo1 channel expression and thereby restrains the rise in the [Ca^2+^]_i_ to facilitate cofilin phosphorylation and filopodia formation, leading to an increase in lung cancer cell migration. These findings broaden our understanding of the molecular mechanism by which the Piezo1 channel functions in lung cancer differently from in other cancers.

## Graphical abstract

**Figure:**
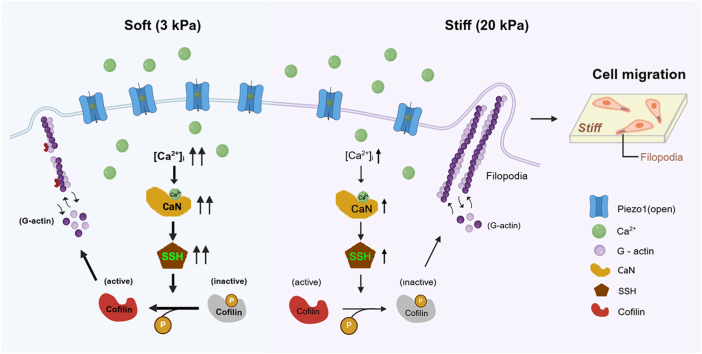

## 1 Introduction

Lung cancer is the leading cause of cancer-related death worldwide, among which non-small cell lung cancers (NSCLC) account for about 85 % [1]. Despite the great advances in medical treatments, the 5-year survival rate of lung cancer remains less than 10 % [2]. Similar to other solid tumors, the biophysical and biochemical cues in the tumor microenvironments in concerted actions influence lung cancer cell functions, including proliferation, migration, invasion as well as epithelial to mesenchymal transition (EMT), and thereby drive cancer progression [[3], [4], [5]]. Therefore, clarifying the molecular mechanism, by which the microenvironmental cues regulate lung cancer cell functions, would assist optimization and development of therapeutic strategies.

Extracellular matrix (ECM) is an important part of the tumor microenvironments, a complex network assembled by a variety of matrix components. Solid tumors are in common characterized by stiffened ECM, with the elastic modulus of cancerous lung (15–100 kPa) being much higher than that of normal lung parenchyma (0.5–5 kPa) [6,7]. It is well established that ECM stiffness strongly influences cancer cell functions, cancer progression and even drug resistance. In general, cells including cancer cells sense, transmit and respond to the changes in matrix stiffness using multiple mechanosensitive machineries, such as integrins, ion channels and cytoskeletal proteins [8]. Piezo1, as an intrinsically mechanosensitive ion channel, is critically engaged in these processes [[9], [10], [11]]. In most cancers, the expression of Piezo1 channel is up-regulated and positively related to cancer cell migration [12]. For example, its knockout inhibited gastric cancer cell invasion and xenograft tumor growth in BALB/c nude mice [13]. Intriguingly, the Piezo1 expression in cancerous lung tissue is lower than that in normal lung tissue, and its knockdown promoted cell migration in both NSCLC and small cell lung cancers (SCLC) models [[14], [15], [16]]. Given the importance of ECM stiffness, interesting questions arise, namely, does the Piezo1 channel respond differently in lung cancer migration, does it still play an inhibitory regulatory role, and what molecular mechanism is responsible for such a unique role?

Usually, cells on stiff substrates reorganize their actin-cytoskeleton for spreading or moving [17]. In some cases, actin-cytoskeletons are assembled and polymerized into special plasma membrane protrusion, named lamellipodia or filopodia [18]. Filopodia are thin finger-like, actin-rich protrusions and play a crucial role in probing ECM stiffness and directing cancer cell migration [19]. There is increasing evidence that filopodia formation and increases in its length and number facilitate cancer cell migration, invasion and metastasis [20,21]. Specifically, it has been demonstrated that substrate stiffness regulates filopodial activities in lung cancer cells [22]. Filopodia formation is tightly controlled by dynamic actin polymerization and depolymerization, which is tightly regulated by various actin-binding proteins (ABPs) [23]. Cofilin, an actin depolymerizing factor, is a small ABP (19–21 kDa) that severs actin polymerization, regulates filopodia formation and participates in cancer cell migration and invasion [[24], [25], [26], [27]]. Cofilin is inactivated by phosphorylation at Ser-3 by kinases such as RhoA/ROCK (Rho-associated kinase) and LIMK (Lin11, Isl-1 and Mec-3), and reactivated by dephosphorylation by phosphatases such as Slingshot [24]. It is well known that the Piezo1 channel functions mainly through mediating extracellular Ca^2+^ influx to raise the [Ca^2+^]_i_ [9,[28], [29], [30]]. Of more interest, it has been reported that a rise in the [Ca^2+^]_i_ can promote cofilin dephosphorylation/activation through calcineurin (CaN)/Slingshot (SSH) signaling pathway [31]. In addition, it has been shown that mechanical stress-induced activation of the Piezo1 channel in chondrocytes tightly associates with actin polymerization through the RhoA/LIMK/cofilin pathway [32]. Therefore, it is interesting to examine whether the Piezo1 channel is engaged in regulating lung cancer cell migration through the Ca^2+^-dependent cofilin/actin polymerization/filopodia formation pathway.

In this study, we investigated the role of Piezo1 channel in regulating the [Ca^2+^]_i_, cofilin phosphorylation and filopodial formation and thereby mediating matrix stiffness regulation of lung cancer cell migration in A549 cells, a NSCLC line, growing on soft and stiff polyacrylamide (PA) hydrogels with stiffnesses close to these of healthy and cancerous lung tissues, respectively, and aim to gain mechanistic understanding of how the Piezo1 channel is involved in the regulation of lung cancer cell migration by ECM.

## 2 Materials and methods

### 2.1 Bioinformatics data analysis

The public databases were analyzed to compare the Piezo1 expression between cancerous and normal tissues. The clinical data of cancer patients were collected from Gene Expression database of Normal and Tumor tissues 2 (GENT2) and GEO database (NCSLC and normal lung tissues, GEO: GSE18842, GSE19188, GSE63074; pancreatic tumor and normal pancreas tissues, GEO: GSE16515, GSE15471, GSE2361, GSE19650, GSE22780, GSE32688, GSE15932; breast cancer and normal breast tissues, GEO: GSE31448, GSE7904, GSE45827, GSE2109, GSE54002, GSE5764, GSE7307, GSE73613, GSE42568, GSE65216; brain cancer and normal brain tissues, GEO: GSE68848, GSE7307; liver cancer and normal liver tissues, GEO: GSE41804, GSE40873, GSE62232, GSE45436; ovarian cancer and normal ovary tissues, GEO: GSE14764, GSE23603, GSE26712, GSE38666, GSE7307, GSE14407, GSE27651, GSE28044, GSE52460, GSE69428, GSE33805).

### 2.2 Fabrication of polyacrylamide (PA) gels

PA gels were fabricated as previously described [33]. Briefly, mixed solutions were prepared containing 40 % acrylamide stock solution, 2 % bis-acrylamide stock solution, 10 % ammonium persulfate (APS), and tetramethylethylenediamine (TEMED, #HC001L, YTHX, China). The mechanical properties of the gels were modulated by altering the volume of 40 % acrylamide and 2 % bis-acrylamide in the pre-polymerization solution. Gels with distinct stiffness values were fabricated by adjusting the final concentrations of acrylamide (4 %, 10 % and 8 %) and bis-acrylamide (0.3 %, 0.1 % and 0.264 %) to achieve elastic moduli of 3.24 kPa, 10.61 kPa and 19.66 kPa (3 kPa, 10 kPa and 20 kPa in short), which correspond to the stiffness ranges of normal lung parenchyma (0.5–5 kPa), pulmonary fibrosis or early-stage tumors (∼10 kPa) and solid lung tumors (20–30 kPa), respectively [7,33,34]. Then, the PA hydrogels were incubated with 1 mg/mL sulfo-SANPAH (#C1111, ProteoChem, USA) and were activated by exposure to UV (365 nm). Before use, the PA gels were coated with 0.12 mg/mL rat tail collagen type I (#354236, CORNING, USA) overnight at room temperature.

### 2.3 Cell culture and treatment

Two human non-small lung cancer cell lines, A549 and H460, were used in the study and they were obtained from the American Type Culture Collection (ATCC) and cultured in 1640 medium (#11875119, Gibco, USA) containing 10 % fetal bovine serum (FBS, #11011–8611, Sijiqing, China), 100 U/ml penicillin and 100 μg/ml streptomycin (#15140122, Gibco, USA) at 37 °C in a 5 % CO_2_ incubator.

Cells were seeded on PA substrates for the following determinations. In some cases, cells were cultured in medium containing 2 μM Yoda1 (#M9372, AbMole, USA) to activate the Piezo1 channel, 2.5 μM GsMTx4 (#HY-P1410, MedChemExpress, USA) to block the Piezo1 channel, 25 μM BAPTA-AM (#M4973, AbMole, USA) to chelate intracellular Ca^2+^ or adding 2 mM CaCl_2_ to observe extracellular Ca^2+^ influx, 4 μM cyclosporine A (CsA) (#M1831, AbMole, USA) to inhibit CaN, 0.5 μM dasatinib (Das, #9052S, Cell signaling technology) to inhibit YAP nuclear translocation, as well as 10 μM PF-573228 (PF, #HY-10461, MCE) to inhibit focal adhesion kinase (FAK) activation, respectively.

### 2.4 F-actin staining and confocal microscopy

Cells were seeded into 6‐well plates with PA hydrogels-covered glass coverslips at a density of 2 × 10^4^ cells/well, and cultured further for 48 h. Following fixation with 4 % paraformaldehyde and permeabilization with 0.1 % Triton-X, cells were incubated with rhodamine-labeled phalloidin (1:100 dilution, #C2207S, Beyotime, China) for 40 min. Hoechst 33342 (1:1000, #H3570, ThermoFisher, USA) was used to stain nuclei. Images were captured using a confocal laser scanning microscope (ZEISS LSM 880, Germany). The length and number of filopodia were calculated by Image J.

### 2.5 Transwell assay

Cells were seeded on PA substrates at a density of 1 × 10^5^ cells/well in 6-well plates and cultured for 48 h, and cell migration was determined using 24-well transwell chambers (8.0-μm pores; Corning, New York, USA) as previously described [35]. Briefly, 1 × 10^4^ cells in 200 μl serum-free medium were added into the upper transwell chamber, the lower chamber was filled with 500 μl 10 % FBS-containing medium. After incubation for 18 h, the inserts were fixed with 4 % paraformaldehyde and stained with 1 % Crystal Violet (#C616390, Aladdin, China). The cells on upper surfaces of the inserts were swabbed gently. The migrated cells were imaged using a microscope (Nikon Ti2) in 4 random view fields and quantified by counting the number of cells with Image J. The experiments were repeated three times. In each time, there were two replicated samples for each group. Cell migration was calculated by the migrated cell number in the test group normalized to that in control group (as defined in figures).

### 2.6 Western blotting

Cells were seeded on PA substrates as mentioned above. After 48 h in culture, cells were collected and lysed in RIPA buffer (#P0013B, Beyotime, China) containing 1 % protease inhibitor ST507 (#P1050, Beyotime, China). Protein concentrations were determined using a bicinchoninic acid assay kit (#U8915, TIANGEN, China). Twenty micrograms of proteins were separated in 10 % SDS-PAGE (#HC015, YTHX, China) and transferred to PVDF membranes (#BS-PVDF-45, Biosharp, China). Membranes were blocked with 5 % bovine serum albumin (BSA, #ST025, Beyotime, China) in Tris-buffered saline and Tween 20 (TBST, #HE011, YTHX, China) for 1 h and then incubated with primary antibody at 4 °C overnight. The following primary antibodies were used: rabbit anti-Piezo1 antibody (1:100 dilution, #DF12083, Affinity Biosciences, China) and rabbit anti-β-actin antibody (1:500 dilution, #GB11001-100, Servicebio, China), anti-p-cofilin and anti-cofilin antibodies (1:100 dilution, #SC365882 and #SC376476, Santa, USA). After three washes with TBST, membranes were incubated with secondary horseradish peroxidase-conjugated anti-rabbit IgG antibody (1:5000 dilution, #ZB2301, Origene, China) for 1 h at room temperature. After extensively washed in TBST, protein bands were visualized using enhanced chemiluminescence (#AR1197, BOSTER, USA) and images captured by MiniChemi (Tanon-4800, Tanon, China) and analyzed with Image J. β-actin was used as loading control.

### 2.7 Flow cytometry

The protein expressions of Piezo1 on cell surface and the level of phospho-SSH1 (p-SSH1) were determined by flow cytometry as previously described [36]. Briefly, cells were harvested and fixed with 4 % paraformaldehyde for 30 min, then permeabilized with 0.3 % Triton X-100 for 10 min. After that, cells were incubated with the primary polyclonal antibody recognizing the extracellular domain of Piezo1 protein (1:100 dilution, #15939–1-AP, Proteintech, USA) or p-SSH1 (p Ser978) (1:100 dilution, #NBP3-23411, Novus Biologicals) for 40 min. Then, cells were incubated with FITC-labeled goat anti-rabbit secondary antibody solution (1:100 dilution, #A0562, Beyotime, China) at room temperature for 1 h away from light. Cells were incubated with the same secondary antibody as negative control (NC). The fluorescence intensity was detected by flow cytometer (BD Accuri C6). The protein expression was derived by subtracting the fluorescence intensity of NC from the fluorescence intensity of specific binding. All data were analyzed using FlowJo software.

### 2.8 Calcium imaging

Changes in the [Ca^2+^]_i_ were monitored by single-cell Ca^2+^ imaging as previously described [37]. Briefly, 2 × 10^4^ cells were seeded on 32-mm coverslips with PA hydrogels and cultured in 10 % FBS-containing RPMI 1640 medium with or without 2.5 μM GsMTx4 for 48 h. After that, cells were washed twice with Ca^2+^-containing buffer (10 mM HEPES, 140 mM NaCl, 2 mM CaCl_2_, 1.13 mM MgCl_2_, 4.7 mM KCl, 10 mM glucose) and loaded with 2.5 μM Fluo4-AM (#F14201, ThermoFisher, USA) for 30 min at room temperature. To investigate Piezo1-mediated extracellular Ca^2+^ influx, cells was imaged firstly in Ca^2+^-free buffer for 1 min and then in buffer containing 2 mM CaCl_2_. Images of fluorescence excited at 488 nm and emitted at 515 nm–3 random fields were captured using a confocal microscope (Andor, Dragonfly). The Fluo4 fluorescence intensity in individual cells was analyzed using Image J.

### 2.9 CaN activity determination

The changes in the CaN activity in A549 cells after indicated treatments were determined using CaN Phosphatase Activity Assay Kit (#ab139461, Abcam), according to the instruction's protocol. Briefly, the cell lysates were mixed with the CaN assay buffer, followed by addition of CaN substrates and incubation for further 10 min. Then, the green assay reagent was added into the mixture, and the OD value at 620 nm was recorded using a microplate reader (Thermo Fisher Scientific). The CaN activity in each group was calculated relative to that in the 3 kPa group.

### 2.10 Data presentation and statistical analysis

All data are presented as means ± SD, where appropriate. Student's t-test was performed for comparison of two groups, and one-way analysis of variance (ANOVA) followed by post hoc Fisher's test for comparison of three or more groups, with p < 0.05 being considered significant.

## 3 Results

### 3.1 The Piezo1 channel expression profile in lung cancer is opposite to that in other cancers

**Fig. 1:**
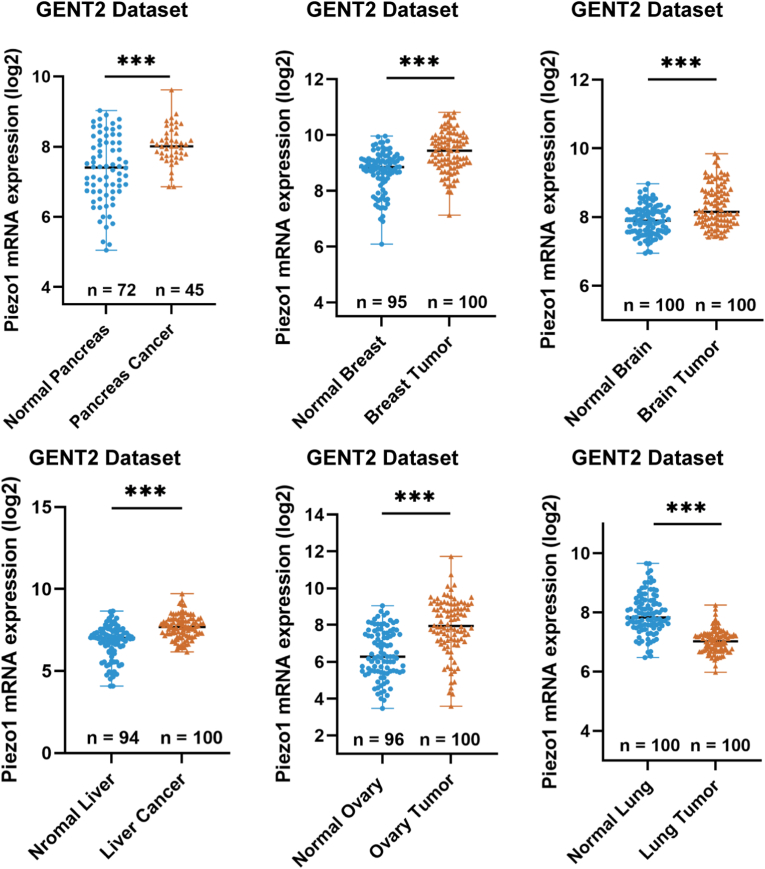
The Piezo1 expression profile in ovary tumor, breast tumor, brain tumor, liver tumor, ovary tumor, lung tumor and matched healthy tissues, based on analysis of GENT2 datasets. Data were presented as the mean ± SD. ∗∗∗P < 0.001.

There are only three publications directly researching the association between Piezo1 channel and lung cancer cell migration [[14], [15], [16]]. We analyzed the Piezo1 expression in six types of solid tumors using the GENT2 public datasets. As shown in Fig. 1, the Piezo1 expression was significantly up-regulated in pancreas cancer, breast cancer, brain cancer, liver cancer and ovary cancer. In contrast, the Piezo1 expression was significantly down-regulated in lung cancer. These results show that the expression of the Piezo1 channel in lung cancer is opposite to that in other types of solid tumors.

### 3.2 Stiff matrix promotes cell migration and downregulates the Piezo1 channel expression

**Fig. 2:**
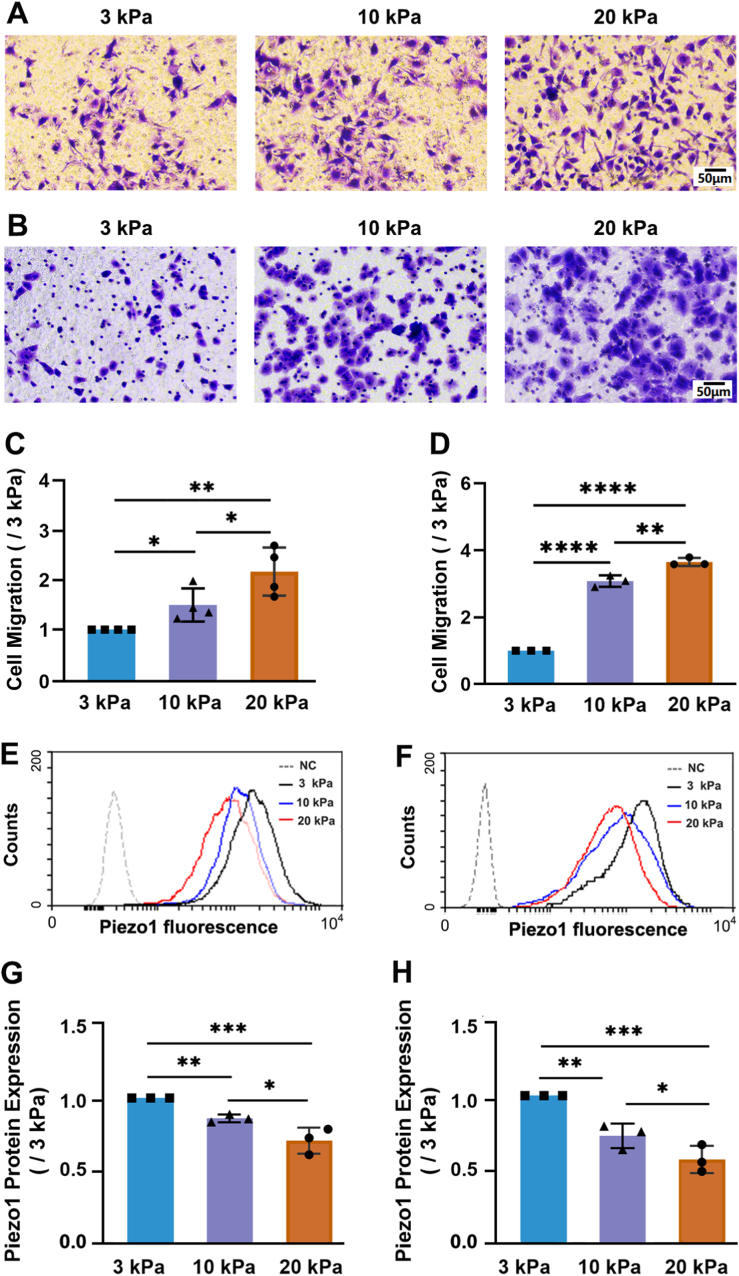
Stiff substrate promotes A549 and H460 cell migration and down-regulates Piezo1 channel e**xpression.** (A–D) Transwell assay of the effects of substrate stiffness on cell migration. Representative images of migrated cells stained with crystal violet (10x, A-B) and statistical analysis of data from three independent experiments (C–D). Scale bar: 50 μm. (E–H) Flow cytometry assessing the effects of substrate stiffness on cell surface Piezo1 protein expression. Representative images of flow cytometry (E–F) and statistical analysis of data from three (G–F) independent experiments. All data were normalized to that of 3 kPa group. Data were presented as mean ± SD. ∗*P* < 0.05; ∗∗*P* < 0.01; ∗∗∗*P* < 0.001; ∗∗∗*P* < 0.0001. (For interpretation of the references to colour in this figure legend, the reader is referred to the Web version of this article.)

To investigate the effects of matrix stiffness on lung cancer cell migration and the Piezo1 channel expression, we prepared PA gels with stiffness close to that of reported healthy (3 kPa, soft) or cancerous (10 and 20 kPa, stiff) lung tissues and cultured A549 or H460 cells on them (Fig. S1). Transwell assay showed that A549 cells on the 10 and 20 kPa substrates migrated significantly faster than those on the 3 kPa substrate (Fig. 2A and C). Flow cytometry (Fig. 2E and G) and Western blotting (Fig. S2) demonstrated that the levels of both membrane-bound and total Piezo1 channel were significantly lower in A549 cells on the 10 and 20 kPa substrate than on the 3 kPa substrate. Furthermore, H460 cells showed a similar increase in cell migration but decrease in the Piezo1 protein expression on the 10 and 20 kPa substrates in comparison with that on 3 kPa substrate (Fig. 2B–D, F and H). Interestingly, cell migration or the Piezo1 expression exhibited substrate stiffness-dependent increase or decrease. Collectively, these results suggest that stiff substrates promote cell migration but downregulates the Piezo1 channel expression.

### 3.3 Downregulation of Piezo1 channel expression is crucial for stiff substrate-induced cell migration

**Fig. 3:**
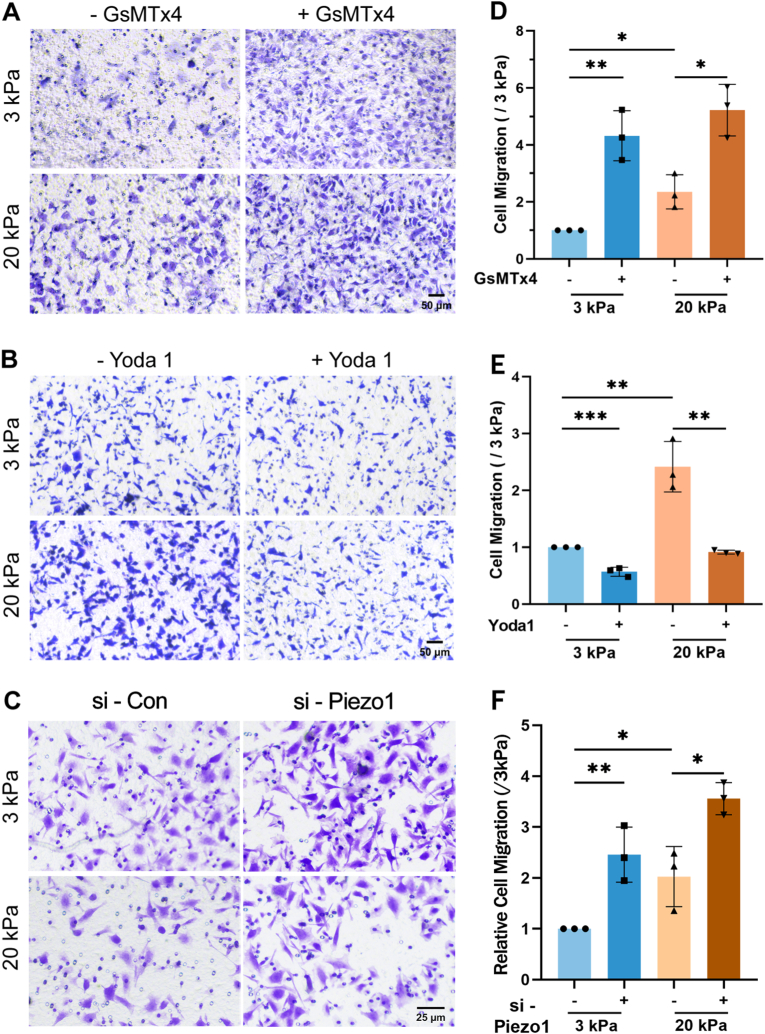
Piezo1 channel negatively regulates substrate stiffness-induced A549 cell **migration.** (A, D) Piezo1 channel blockade with GsMTx4 promotes cell migration on both soft and stiff substrates. (B, E) Piezo1 channel activation with Yoda 1 inhibits cell migration on both soft and stiff substrates. (C, F) Piezo1 channel knockdown with specific siRNA transfection promotes cell migration on both soft and stiff substrates. Representative images of migrated cells stained with crystal violet (10x, A-C) and statistical analysis of data from three independent experiments (D–F). Scale bar: 50 μm. All data were normalized to the 3 kPa group. Data were presented as mean ± SD. ∗p < 0.05, ∗∗p < 0.01, ∗∗∗p < 0.001. (For interpretation of the references to colour in this figure legend, the reader is referred to the Web version of this article.)

To investigate the linkage between the increased cell migration and the decreased Piezo1 expression, we next examined cell migration in the absence or presence of Piezo1 channel blockade or activation. Treatment with GsMTx4 to block the Piezo1 channel significantly promoted A549 cell migration on both soft and stiff substrates (Fig. 3A and D). In contrast, exposure to Yoda1 induced Piezo1 channel activation significantly inhibited cell migration, with a greater inhibition in cells on the stiff substrates (Fig. 3B and E). We further investigated the effect of Piezo1 knockdown on matrix stiffness-regulated cell migration. Treatment with Piezo1-specific siRNA strongly reduced the Piezo1 expression (Fig. S3), and promoted A549 cell migration on both soft and stiff substrates (Fig. 3C and F). Similarly, the channel blockade with GsMTx4 treatment promoted, but the channel activation with Yoda-1 treatment inhibited H460 cell migration (Fig. S4). These results indicate that the Piezo1 channel plays a negative regulatory role in substrate stiffness-induced lung cancer cell migration.

### 3.4 Downregulation of Piezo1 channel expression facilitates stiff substrate-induced filopodia formation

**Fig. 4:**
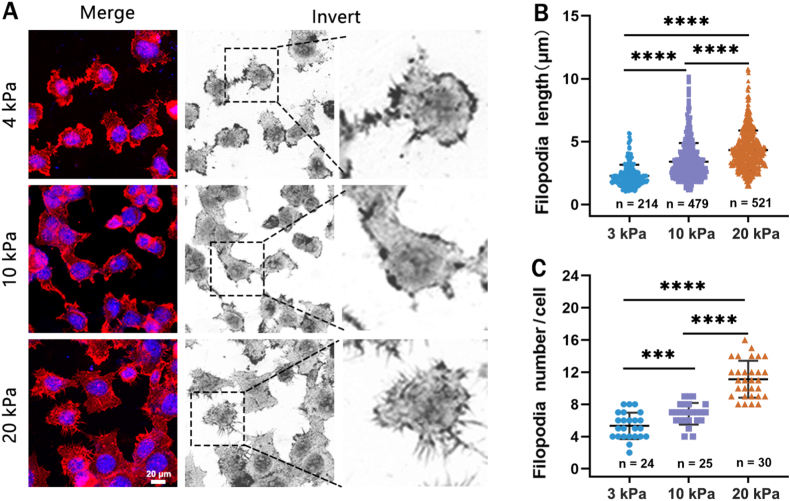
Filopodia formation in A549 cells on 3, 10 and 20 kPa substrates, respectively. Representative images of filopodia morphology (A) and statistical analysis of the filopodia length (B) and number (C) from indicated number of cells. Red, F-actin staining with rhodamine-labeled phalloidin; blue, nucleus staining with Hoechst 33342. All data were normalized to that of the 3 kPa group. Scale bar: 20 μm. Data were presented as mean ± SD. ∗∗∗P < 0.001; ∗∗∗∗P < 0.0001. (For interpretation of the references to colour in this figure legend, the reader is referred to the Web version of this article.)

**Fig. 5:**
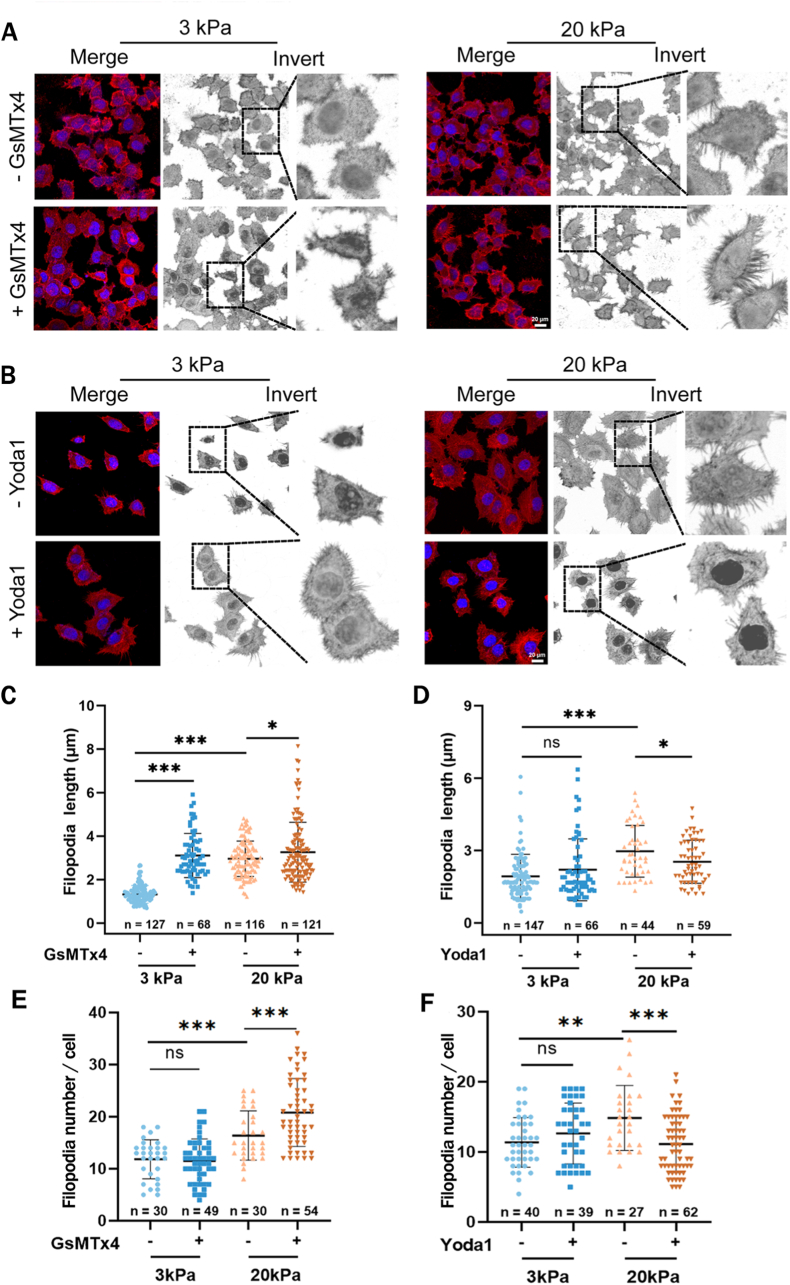
Piezo1 channel negatively regulates stiff substrate-induced filopodia formation in A**549 cells.** (A, C, D) Piezo1 channel blockade with GsMTx4 further promotes filopodia formation in cells on both soft and stiff substrates. (B, E, F) Piezo1 channel activation with Yoda 1 further inhibits filopodia formation in cells on stiff substrates but has no effect in cells on soft substrates. Representative images of filopodia morphology (A and B) and statistical analysis of the filopodia length (C and E) and number (D and F) from indicated number of cells. Red, F‐actin staining with rhodamine-labeled phalloidin; blue, nucleus staining with Hoechst 33342. All data were normalized to that of the 3 kPa group. Scale bar: 20 μm. Data were presented as mean ± SD. ∗*P* < 0.05; ∗∗∗*P* < 0.001; ns, not significant. (For interpretation of the references to colour in this figure legend, the reader is referred to the Web version of this article.)

To clarify how the downregulated Piezo1 channel contributes to stiff substrate-induced increase in A549 cell migration, we focused on filopodium formation, due to its implication in probing ECM stiffness and directing cancer cell migration [20]. More importantly, it has been reported that filopodia formation is negatively associated with the Piezo1 channel expression in Hela cells [38]. We firstly examined the filopodia formation of A549 cells seeding on the 3, 10 and 20 kPa substrates, respectively. Similar to cell migration and Piezo1 expression, the filopodia formation were promoted in a substrate stiffness-dependent manner, which A549 cells on the 10 and 20 kPa substrates exhibited longer filopodia and more numbers of filopodia than those on the 3 kPa substrate (Fig. 4). We further investigated the role of the Piezo1 channel in such stiff substrate-induced enhancement of filopodia formation by blockade with GsMTx4 or activation with Yoda1. Piezo1 channel blockade did not abolish but promoted filopodia formation on both soft (3 kPa) and stiff (20 kPa) substrates (Fig. 5A–C and E). In contrast, Piezo1 channel activation inhibited filopodia formation on the stiff substrate, without effect on the soft substrate (Fig. 5B–D and F). Consistently, Piezo1 channel blockade or activation led to a promotive or inhibitory effect on stiffness-regulated filopodia formation in H460 cells (Fig. S5). These results indicate that the Piezo1 channel mediates substrate stiffness modulation of filopodia formation, with the down-regulated Piezo1 channel facilitating stiff substrate-induced filopodia formation.

### 3.5 The lower [Ca^2+^]_i_ caused by the down-regulated Piezo1 channel is required for stiff substrate-induced cell migration and filopodia formation

**Fig. 6:**
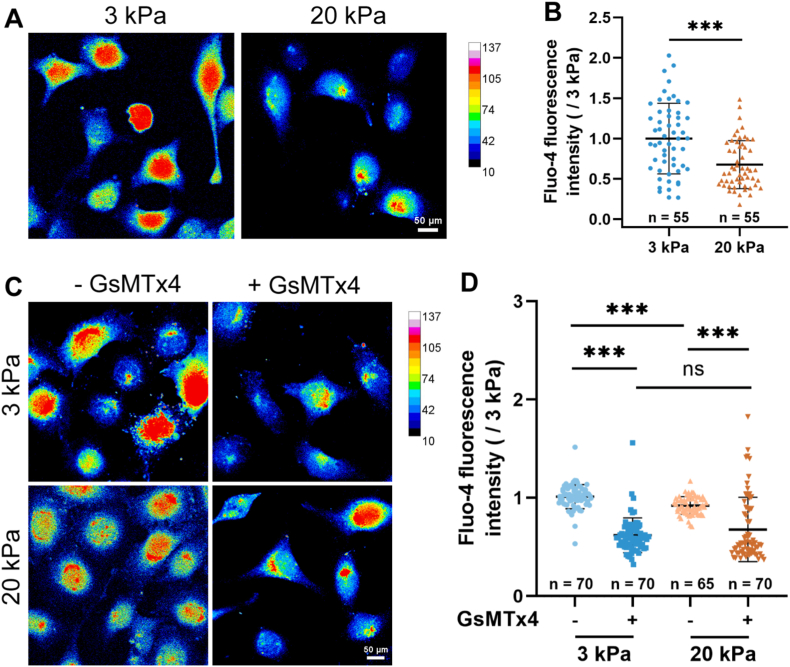
Piezo1 channel mediates substrate stiffness-induced change in [Ca^2+^]_i_ in A**549 cells.** (A, B) Cells showed the higher and lower [Ca^2+^]_i_ in cells on soft and stiff substrates, respectively. (C, D) Piezo1 channel blockade with GsMTx4 reduces [Ca^2+^]_i_ in cells on both soft and stiff substrates. Representative Ca^2+^ images (A, C and E) and statistical analysis of [Ca^2+^]_i_ in indicated numbers of cells (B and D). Scale bar: 50 μm. All data were normalized to that of 3 kPa group. Data were presented as mean ± SD. ∗∗∗*P* < 0.001; ns, not significant.

**Fig. 7:**
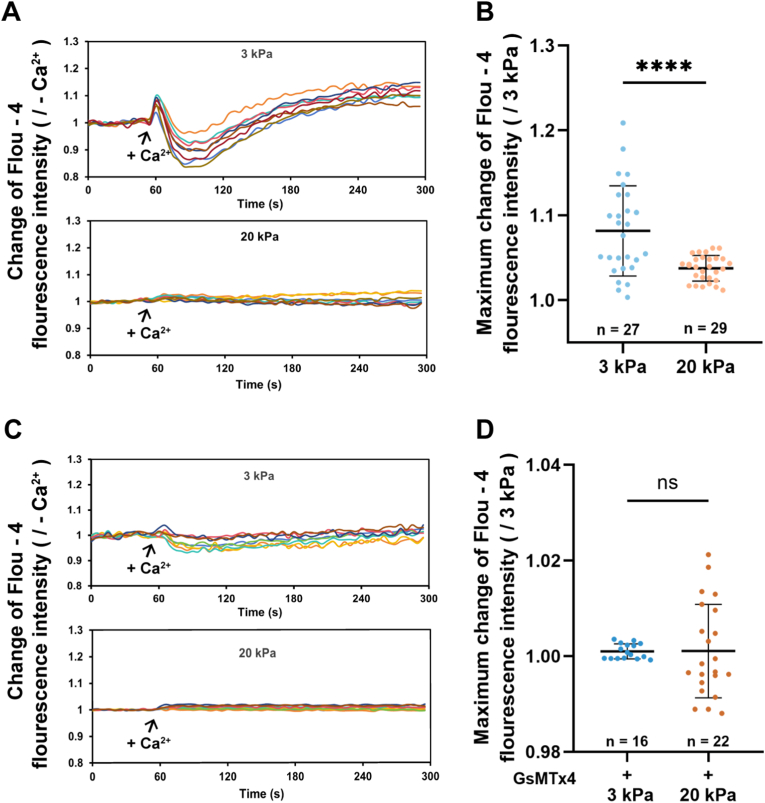
Piezo1 channel mediates a strong but weak calcium influx induced by soft and stiff substrates, respectively. (A and B) Extrcellular Ca^2+^ influx in cells on soft and stiff substrates. (C and D) Piezo1 blockade with GsMTx4 abolished the difference in Ca^2+^ influx between cells on soft and stiff substrates. Representative tracces showing change in [Ca^2+^]_i_ (A and C) and statistical analysis of the maximal change in [Ca^2+^]_i_ in indicated numbers of cells (B and D). Cells were cultured in medium with or without 2.5 μM GsMTx4 containment for 48 h. Cells loaded with Fluo4-AM were imaged with 5 *s* interval in Ca^2+^-free buffer for 1 min and further 4 min upon addition of 2 mM CaCl_2_. Scale bar: 50 μm. All data were normalized to that ones prior to addition of CaCl_2_. Data were presented as mean ± SD. ∗∗∗*P* < 0.001.

Both A549 and H460 cells followed the same patterns in the stiffness regulation of the Piezo1 channel expression and the role of Piezo1 in the stiffness regulation cell migration and filopodia formation and, thus, further experiments were carried out using A549 cells to gain the underlying mechanisms. The Piezo1 channel mainly functions as a Ca^2+^-permeable channel mediating extracellular Ca^2+^ influx to raise intracellular [Ca^2+^]_i_ [9,[28], [29], [30]]. Therefore, to probe the mechanisms by which the down-regulated Piezo1 channel regulates stiff substrate stimulation of A549 cell migration and filopodia formation, we first examined the role of the Piezo1 channel in substrate stiffness-induced changes in the [Ca^2+^]_i_ in A549 cells. As shown in Fig. 6A and B, the [Ca^2+^]_i_ in cells on the stiff substrate was lower than that in cells on the soft substrate. Piezo1 channel blockade reduced the [Ca^2+^]_i_ in A549 cells on both soft and stiff substrates and, as a result, abolished the difference in the [Ca^2+^]_i_ in A549 cells on both substrates (Fig. 6C and D). To further investigate whether this difference in [Ca^2+^]_i_ between cells on soft and stiff substrates, especially the lower [Ca^2+^]_i_ in cells on the stiff substrate, was cause by the Piezo1 channel, we compared extracellular Ca^2+^ influx in cells on the soft and stiff substrates. As shown in Fig. 7A and B, introduction of Ca^2+^ in extracellular solutions induced extracellular influx that was higher or more noticeable in cells on the soft substrates compared to that in cells on the stiff substrates. Such difference in extracellular Ca^2+^ influx was largely abolished by prior treatment with GsMTx4 to block the Piezo1 channel (Fig. 7C and D). These results indicate that downregulation of the Piezo1 channel expression induced by stiff substrate restrains extracellular Ca^2+^ influx, resulting in a lower [Ca^2+^]_i_ in cells on the stiff substrates.

**Fig. 8:**
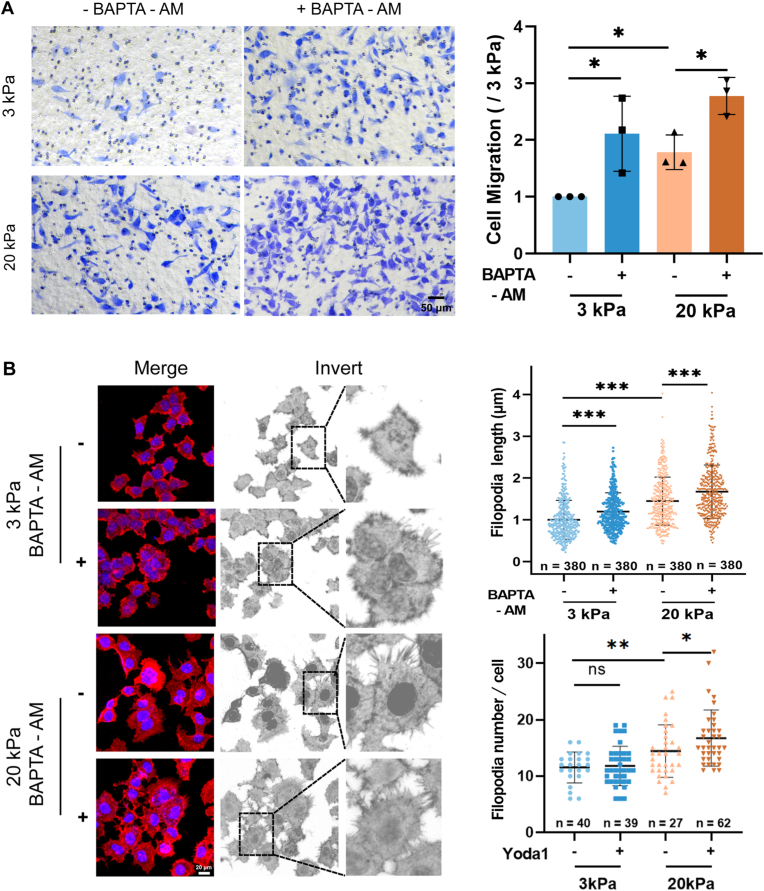
Stiff substrate-induced reduction in [Ca^2+^]_i_ is required for migration and filopodia formation in A**549 cells.** (A) Chelation of intracellular Ca^2+^ with BAPTA-AM promotes A540 cell migrations on both soft and stiff substrates. Representative images of migrating cells stained with crystal violet (10x, left) and statistical analysis of data from three independent experiments (right). Scale bar: 50 μm. (B) Chelation of intracellular Ca^2+^ with BAPTA-AM promotes filopodia formation in A540 cells on both soft and stiff substrates. Representative images of filopodia (left) and statistical analysis of filopodia length and number from the defined filopodia numbers and cell numbers. Scale bar: 20 μm. Red, F‐actin staining with rhodamine-labeled phalloidin; blue, nucleus staining with Hoechst 33342. All data were normalized to that of the 3 kPa group. Data were presented as mean ± SD. ∗*P* < 0.05; ∗∗∗*P* < 0.001. (For interpretation of the references to colour in this figure legend, the reader is referred to the Web version of this article.)

We next investigated whether such Piezo1-mediated reduction in the [Ca^2+^]_i_ contributes to stiff substrate-induced regulation of A549 cell migration and filopodia formation. Treatment with BAPTA-AM, a Ca^2+^ chelator, to reduce the [Ca^2+^]_i_ significantly promoted A549 cell migration on both soft or stiff substrates (Fig. 8A). Similarly, treatment with BAPTA-AM promoted filopodia formation in cells on both soft and stiff substrates (Fig. 8B). Collectively, these results suggest that the [Ca^2+^]_i_ negatively regulates stiff matrix-induced A549 cell migration and filopodia formation, and the lower [Ca^2+^]_i_ due to downregulation of the Piezo1 channel is required for stiff substrate-induced regulation of cell migration and filopodia formation.

### *3.6* *The lower [*Ca^2+^*]*_*i*_*due to the down-regulated Piezo1 channel is indispensable for stiff substrate-induced phosphorylation of cofilin*

**Fig. 9:**
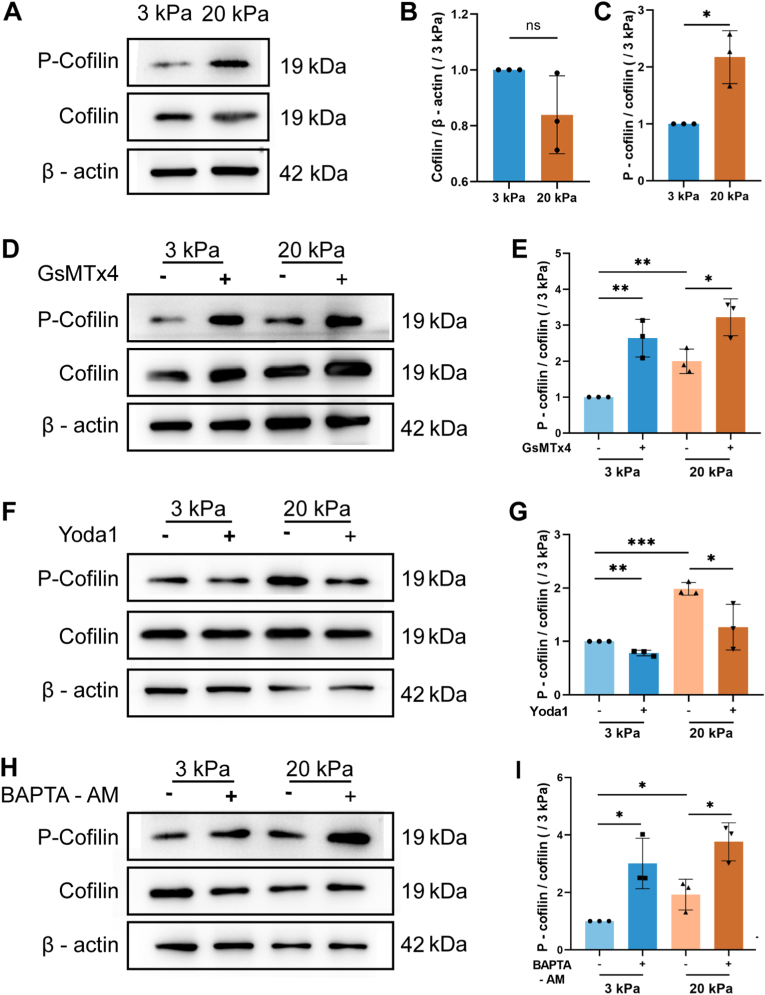
Piezo1 channel regulates stiff substrate-induced phosphorylation of coflilin through reducing the [Ca^2+^]_i_ in A**549 cells.** (A–C) Stiff substrate induces phosphorylation of cofilin (C), without effect on its expression (B). (D–G) Stiff substrate-induced phosphorylation of coflilin is enhanced by Piezo1 channel blockade with GsMTx4 (D and E) but attenuated by Piezo1 channel activation with Yoda-1 (F and G). (H, I) Chelation of intracellular Ca^2+^ with BAPTA-AM promotes cofilin phosphorylation in cells on both soft and stiff substrates. Representative images of western blotting (A, D, F and H) and statistical analysis of data from three independent experiments (B, C, E, G and I). All data were normalized to that of the 3 kPa group. Data are presented as mean ± SD. ∗*P* < 0.05; ∗*P* < 0.01; ∗∗∗*P* < 0.001.

It is reported that the phosphorylated cofilin loses its ability to bind to and cut off F-actin polymerization, thereby facilitating filopodia formation and cancer cell migration [21]. In addition, the status of cofilin phosphorylation is regulated by the [Ca^2+^]_i_ [39,40]. Therefore, to further decipher the downstream molecular mechanisms by which Piezo1 channel-mediated reduction in [Ca^2+^]_i_ modulates stiff substrate-induced filopodia formation and cell migration, we investigated the phosphorylation level of cofilin (p-cofilin) in cells on the soft and stiff substrates with or without treatment with BAPTA-AM, Piezo1 channel blockade and activation. As shown in Fig. 9A–C, compared to the soft substrates, stiff substrates significantly promoted the p-cofilin level. In addition, Piezo1 channel blockage increased, whereas Piezo1 channel activation attenuated, the p-cofilin levels in cells on both soft and stiff substrates, (Fig. 9D–G). Furthermore, the p-cofilin levels in cells on both soft and stiff substrates were further enhanced by treatment with BAPTA-AM, suggesting that decrease in the [Ca^2+^]_i_ favored cofilin phosphorylation (Fig. 9H and I). These results suggest that the [Ca^2+^]_i_ negatively regulates the p-cofilin level and the lower [Ca^2+^]_i_ resulting from the down-regulated Piezo1 expression is indispensable for stiff substrate-induced phosphorylation of cofilin.

### *3.7* *Attenuation of CaN/SSH phosphatase by lower [*Ca^2+^*]*_*i*_*due to the down-regulated Piezo1 channel is required for stiff substrate-induced phosphorylation of cofilin*

**Fig. 10:**
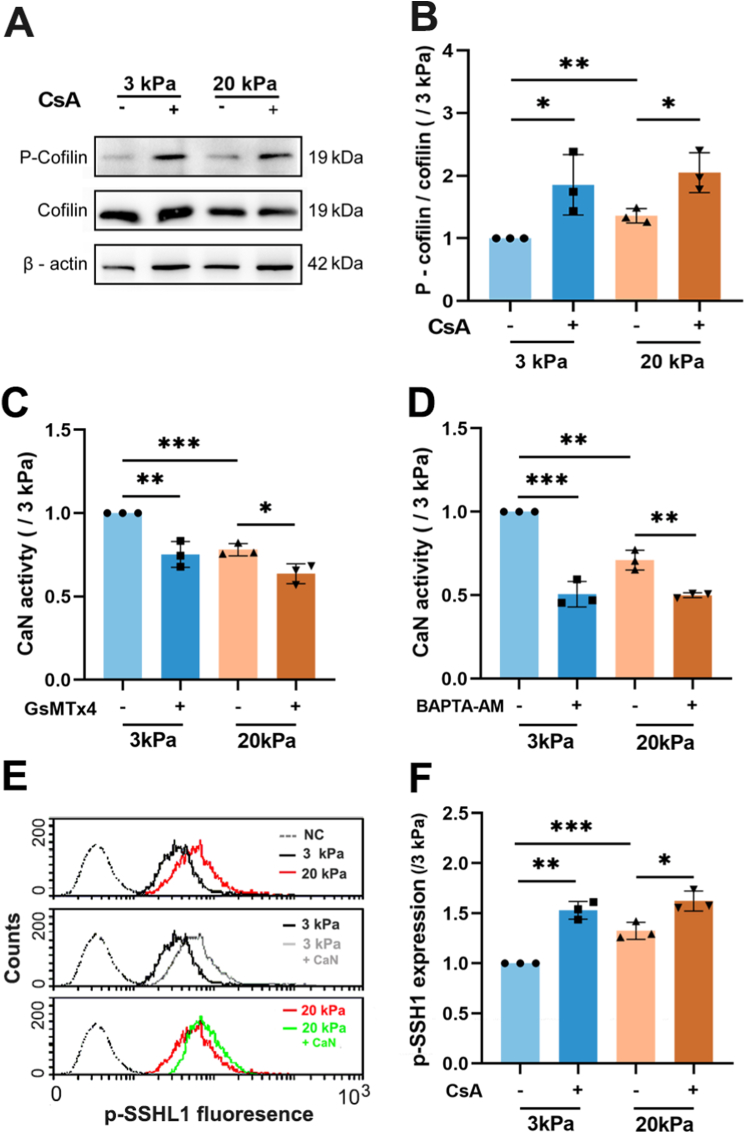
Piezo1 channel regulates stiff substrate-induced phosphorylation of coflilin through attenuating the Ca^2+^-dependent CaN/SSH activation in A**549 cells.** (A–B) CaN inhibition with CsA promotes cofilin phosphorylation in cells on both soft and stiff substrates. (C–D) CaN activity was decreased on stiff substrate, and further decreased on soft and stiff substrates by Piezo1 channel blockade with GsMTx4 (B) or Chelation of intracellular Ca^2+^ with BAPTA-AM (D), respectivley. (E–F) The p-SSH1 was incrased on stiff substrates, and further increased on soft and stiff substrates by CaN inhibition with CsA. Representative images of western blotting (A) and flow cytometry (E), and statistical analysis of data from three independent experiments (B, C, D and F). All data were normalized to that of 3 kPa group. Data are presented as mean ± SD. ∗*P* < 0.05; ∗*P* < 0.01; ∗∗∗*P* < 0.001.

As mentioned in the Introduction, the p-cofilin level is also regulated by the CaN/SSH phosphatase, which is activated by a rise in the [Ca^2+^]_i_, thereby promoting cofilin dephosphorylation and a consequent decrease in the p-cofilin level [31]. Therefore, to further clarify how the lower [Ca^2+^]_i_ mediated by the down-regulated Piezo1 channel on stiff substrates increased the p-cofilin level, we firstly examined the p-cofilin level in cells on the soft and stiff substrates with or without treatment with CsA to inhibit CaN. As shown in Fig. 10A and B, the p-cofilin levels in cells on both soft and stiff substrates were significantly enhanced by treatment with CsA, suggesting an engagement of CaN in stiffness regulation of increase in the p-cofilin level. Next, we compared the CaN activity in cells on the soft and stiff substrates with or without Piezo1 channel blockade or intracellular Ca^2+^ chelation, respectively. As shown in Fig. 10C and D, the CaN activity in cells on the stiff substrate was lower than that in cells on the soft substrate. Piezo1 channel blockade or intracellular Ca^2+^ chelation reduced the CaN activity in cells on both soft and stiff substrates and, as a result, abolished the difference in the CaN activity in A549 cells on both substrates, suggesting linkage of the lower CaN activity with the lower [Ca^2+^]_i_ and the Piezo1 expression on the stiff substrates. We further examined the CaN-dependent SSH1 dephosphorylation by comparing the p-SSH1 level in A549 cells on the soft and stiff substrates with or without CaN inhibition. As shown in Fig. 10E and F, the p-SSH1 level in cells on the stiff substrate was higher than that in cells on the soft substrate, and CaN inhibition reduced the p-SSH1 level in cells on both soft and stiff substrates and, furthermore, abolished the difference in the p-SSH1 level in cells on both substrates, suggesting correlation between substrate stiffness regulation of CaN activity and SSH activity. Collectively, these results suggest that the lower [Ca^2+^]_i_ resulting from the down-regulated Piezo1 expression attenuated the activity of CaN/SSH1, which is required for stiff substrate-induced phosphorylation of cofilin.

## 4 Discussion

Different from what has been observed in most solid tumors, the Piezo1 channel plays a negative regulatory role in lung cancer cell migration and lung cancer metastasis. The present study shows that ECM stiffness is implicated in such negative regulation and, mechanistically, stiff substrates down-regulate the Piezo1 channel expression and restrain the [Ca^2+^]_i_ to favor cofilin phosphorylation and facilitates filopodia formation, thereby promoting lung cancer cell migration.

As a common characteristic of solid tumors, stiffening substrates promotes cancer cell migration, as shown by previous [3,4] and present studies (Fig. 2A–D). However, it remains unknown how the Piezo1 channel in lung cancer cells respond to substrate stiffness and related to stiff substrate-induced lung cancer cell migration. The present study showed that blockade of the Piezo1 channel or siRNA-mediated knockdown of its expression promoted cell migration, whereas activation resulted in an inhibitory effect (Fig. 3 and Fig. S4). These results demonstrate that the Piezo1 channel is engaged in stiff substrate-induced lung cancer cell migration in a negative manner. Similar to our findings, Pathak and colleagues reported that knockout of the Piezo1 channel expression promoted, whereas the Piezo1 channel activation attenuated keratinocyte migration in skin wound healing, where the skin wound and skin scar are stiff compared to normal skin tissues [41].

Filopodia is crucial in probe probing ECM stiffness and directing cancer cell migration [42]. In this study, we showed that the downregulated Piezo1 channel expression in A549 and H460 cells on the stiff substrate enhanced filopodia formation. In contrast, the higher Piezo1 channel expression in cells on the soft substrates resulted in less filopodia formation (Fig. 2, Fig. 4). Furthermore, Piezo1 channel blockade exerted a stimulatory effect and, by contrast, channel activation exerts an inhibitory effect on filopodia formation on the stiff substrate (Fig. 5 and Fig. S5). Yang et al. have demonstrated that Piezo1 overexpression in HEK239T cells inhibited filopodia formation and, as reported in their study, the Piezo1 channel is depleted in highly curved membrane protrusions such as filopodia and enriched to nanoscale membrane invaginations [38]. Our results are consistent with those by Yang et al. and together highlights an important role of the Piezo1 channel in substrate-induced negative regulation of stiff lung cancer cell migration through modulating filopodia formation.

Piezo1 channel functions by mediating extracellular Ca^2+^ influx to raise the [Ca^2+^]_i_ [9,[28], [29], [30]] and the [Ca^2+^]_i_ is critical in regulating cell migration through modulating filopodia formation [40,43,44]. We showed that Piezo1 channel blockade attenuated the [Ca^2+^]_i_ in cells on both soft and stiff substrates (Fig. 6). Particularly, we showed that it is through downregulating the Piezo1 channel expression that resulted in the lower [Ca^2+^]_i_ in cells on the stiff substrates; there were higher extracellular Ca^2+^ influx in cells on the soft substrate than in cells on the stiff substrate, and Piezo1 channel blockade abolished the difference (Fig. 7). Accordingly, treatment with BAPTA-AM to reduce the [Ca^2+^]_i_ produced a stimulatory effect on lung cancer migration on both soft and stiff substrate (Fig. 8A). Similar to the effects on cell migration, reduction in the [Ca^2+^]_i_ also promoted filopodia formation in cells on both soft and stiff substrates (Fig. 8B). Our results are consistent to that from a previous report that Ca^2+^ overload blunts filopodia formation and inhibits cell migration in endothelial cells [40] and provide evidence to suggest that the Piezo1 channel negatively regulates stiff substrate-induced lung cancer cell migration through reducing the [Ca^2+^]_i_ and facilitating filopodia formation.

It is well established that the status of cofilin phosphorylation is crucial for actin reorganization, especially filopodia formation, and thereby cancer cell migration [[45], [46], [47], [48], [49], [50], [51]]. It has also been reported that the status of cofilin phosphorylation is regulated by the Ca^2+^-dependent CaN/SSH phosphatase pathway [31]. However, the relationships remain unknown among the Piezo1 channel, [Ca^2+^]_i_, CaN, SSH, cofilin phosphorylation and filopodia formation in cancer cell migration, especially in substrate stiffness regulation of cell migration. Our results show that stiff substrate downregulated the Piezo1 channel expression and thereby reduced the [Ca^2+^]_i_ to attenuate CaN/SSH1-mediated cofilin dephosphorylation, promote A549 cell migration, filopodia formation and cofilin phosphorylation (Fig. 2, Fig. 3, Fig. 4, Fig. 5, Fig. 6, Fig. 7, Fig. 8, Fig. 9, Fig. 10). Our finding is consistent with a recent study showing that Piezo1 overexpression in HEK239T cells promoted cofilin dephosphorylation and F-actin filaments severance [52]. However, further investigations are required to clarify how Piezo1 channel-dependent reduction in [Ca^2+^]_i_ promotes cofilin phosphorylation. In addition, the current results and mechanistic explanation are from A549 and H460 cells and it is interesting to evaluate the findings from this study in another lung cancer lines or patient-derived cells in future. We also try to ascertain how stiff substrates downregulate the Piezo1 channel expression, focused on the canonical mechano-transduction pathways, integrin-FAK and Hippo-YAP pathway, and found that the two pathways are not engaged in stiffness downregulation of the Piezo1 channel expression in A549 cells (Fig. S6). These results are to some extent consistent with the previous findings, which depletion of the Piezo1 expression promoted lung cancer cell migration in an integrin-independent manner [15], and Piezo1 acts as the upstream but not downstream molecular of YAP [53,54]. Therefore, it is also interesting to identify the signaling pathway engaged in stiff substrate downregulation of the Piezo1 channel expression in future work.

## 5 Conclusions

**Fig. 11:**
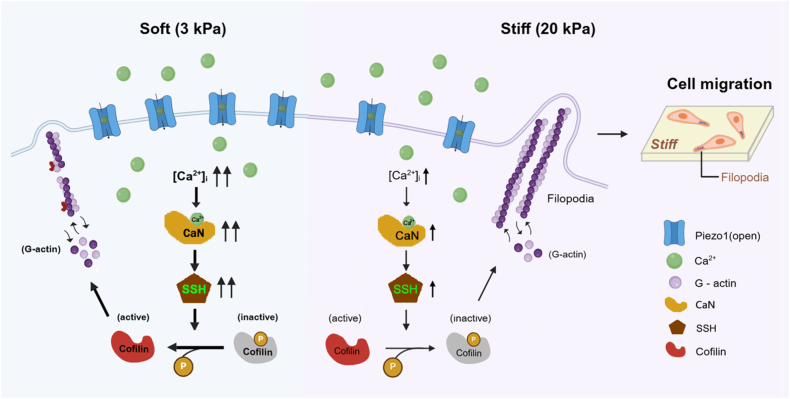
Schematic summary of the Piezo1/calcium/CaN-SSH/cofilin/filopodia formation pathway for substrate stiffness-induced cancer cell **migration.** Stiff matrix downregulates the expression of Piezo1 channel, which limits the [Ca^2+^]_i_ rise and the activity of CaN-SSH. Consequently, cofilin is phosphorylated and inactivated and thereby loses its potential to bind to and sever actin filaments, facilitates filopodia formation, and thereby promote cancer cell migration.

In summary, our study has revealed a negative regulatory role of the Piezo1 channel in mediating stiff matrix-induced lung cancer cell migration (Fig. 11). Mechanistically, stiff substrates downregulate the Piezo1 channel expression and thereby reduces Piezo1 channel-mediated extracellular Ca^2+^ influx to restrain an increase in [Ca^2+^]_i_, which favors phosphorylation/inactivation of cofilin through attenuating CaN/SSH1-mediated cofilin dephosphorylation and facilitates filopodia formation, leading to lung cancer cell migration on stiff substrates. Clinical data from Public datasets (Fig. 1) and experimental data from limited researches [[14], [15], [16]] suggest that the expression of Piezo1 channel is decreased in lung cancer and negatively correlated with lung cancer metastasis, but the underlying mechanism is unknown. The findings described in this study provide a previously unrecognized mechanistic explanation and broaden our understanding of the molecular mechanism how the Piezo1 channel functions in lung cancer and provided opportunities for the development of new lung cancer treatment.

## CRediT authorship contribution statement

**Xiaoling Jia:** Writing – original draft, Supervision, Resources, Methodology, Conceptualization. **Lin Zhao:** Writing – review & editing, Formal analysis, Data curation. **Juncheng Bai:** Methodology, Data curation. **Lu Wen:** Methodology, Data curation. **Qianyu Meng:** Methodology. **Haikun Wang:** Data curation. **Junqi Men:** Methodology. **Hui Shao:** Methodology. **Yingying Guo:** Methodology. **Xinlan Chen:** Data curation. **Xing Chen:** Resources. **Lin-Hua Jiang:** Writing – review & editing, Supervision. **Yubo Fan:** Writing – review & editing, Supervision. **Huawei Liu:** Conceptualization, Funding acquisition, Resources.

## Statement of significance

This is the first study that reports a negative regulatory role of the Piezo1 channel in matrix stiffness regulation of lung cancer cell migration and the molecular mechanism involved. We have revealed that stiff matrix down-regulates the Piezo1 channel expression and thereby maintain a low [Ca^2+^]_i_ to favor cofilin phosphorylation and facilitate filopodia formation, consequently, promoting cell migration. These findings broaden our understanding of the molecular mechanism how the Piezo1 channel functions differently in lung cancer from in most cancers, and provide new strategies for lung cancer treatment.

## Declaration of competing interest

The authors declare no competing financial interests or personal relationships that influence the work reported in this paper.

## Data Availability

All data reported in this study are available upon reasonable requirement.

## References

[1] Siegel R.L., Miller K.D., Wagle N.S., Jemal A. (2023). Cancer statistics. CA Cancer J. Clin..

[2] Guo L.W., Lyu Z.Y., Meng Q.C., Zheng L.Y., Chen Q., Liu Y., Xu H.F., Kang R.H., Zhang L.Y., Cao X.Q., Liu S.Z., Sun X.B., Zhang J.G., Zhang S.K. (2022). A risk prediction model for selecting high-risk population for computed tomography lung cancer screening in China. Lung Cancer.

[3] Jiang Y., Zhang H., Wang J., Liu Y., Luo T., Hua H. (2022). Targeting extracellular matrix stiffness and mechanotransducers to improve cancer therapy. J. Hematol. Oncol..

[4] Najafi M., Farhood B., Mortezaee K. (2019). Extracellular matrix (ECM) stiffness and degradation as cancer drivers. J. Cell. Biochem..

[5] Alonso-Nocelo M., Raimondo T.M., Vining K.H., López-López R., de la Fuente M., Mooney D.J. (2018). Matrix stiffness and tumor-associated macrophages modulate epithelial to mesenchymal transition of human adenocarcinoma cells. Biofabrication.

[6] Asano S., Ito S., Takahashi K., Furuya K., Kondo M., Sokabe M., Hasegawa Y. (2017). Matrix stiffness regulates migration of human lung fibroblasts. Phys. Rep..

[7] Miyazawa A., Ito S., Asano S., Tanaka I., Sato M., Kondo M., Hasegawa Y. (2018). Regulation of PD-L1 expression by matrix stiffness in lung cancer cells. Biochem Bioph Res Co.

[8] Nigro E.A., Boletta A. (2021). Role of the polycystins as mechanosensors of extracellular stiffness. Am J Physiol-Renal.

[9] Coste B., Mathur J., Schmidt M., Earley T.J., Ranade S., Petrus M.J., Dubin A.E., Patapoutian A. (2010). Piezo1 and Piezo2 are essential components of distinct mechanically activated cation channels. Science.

[10] Kim S.E., Coste B., Chadha A., Cook B., Patapoutian A. (2012). The role of Piezo in mechanical nociception. Nature.

[11] Murthy S.E., Dubin A.E., Patapoutian A. (2017). Piezos thrive under pressure: mechanically activated ion channels in health and disease. Nat. Rev. Mol. Cell Biol..

[12] Zhao F., Zhang L., Wei M., Duan W., Wu S., Kasim V. (2022). Mechanosensitive ion channel PIEZO1 signaling in the Hall-Marks of cancer: structure and functions. Cancers (Basel).

[13] Wang X.F., Cheng G., Miao Y., Qiu F.Y., Bai L.G., Gao Z.F., Huang Y.N., Dong L.R., Niu X., Wang X., Li Y.Y., Tang H., Xu Y.Y., Song X.D. (2021). Piezo type mechanosensitive ion channel component 1 facilitates gastric cancer omentum metastasis. J. Cell Mol. Med..

[14] Huang Z., Sun Z., Zhang X., Niu K., Wang Y., Zheng J., Li H., Liu Y. (2019). Loss of stretch-activated channels, PIEZOs, accelerates non-small cell lung cancer progression and cell migration. Biosci. Rep..

[15] McHugh B.J., Murdoch A., Haslett C., Sethi T. (2012). Loss of the integrin-activating transmembrane protein Fam38A (Piezo1) promotes a switch to a reduced integrin-dependent mode of cell migration. PLoS One.

[16] Bo H., Wu Q., Zhu C., Zheng Y., Cheng G., Cui L. (2024). PIEZO1 acts as a cancer suppressor by regulating the ROS/Wnt/β-catenin axis. Thorac. Cancer.

[17] Pollard T.D., Cooper J.A. (2009). Actin, a central player in cell shape and movement. Science.

[18] Heckman C.A., Plummer H.K. (2013). Filopodia as sensors. Cell. Signal..

[19] Arjonen A., Kaukonen R., Ivaska J. (2011). Filopodia and adhesion in cancer cell motility. Cell Adhes. Migrat..

[20] Jacquemet G., Hamidi H., Ivaska J. (2015). Filopodia in cell adhesion, 3D migration and cancer cell invasion. Curr. Opin. Cell Biol..

[21] Aikemu B., Shao Y., Yang G., Ma J., Zhang S., Yang X., Hong H., Yesseyeva G., Huang L., Jia H., Wang C., Zang L., Sun J., Zheng M. (2021). NDRG1 regulates Filopodia-induced colorectal cancer invasiveness via modulating CDC42 activity. Int. J. Biol. Sci..

[22] Liou Y.R., Torng W., Kao Y.C., Sung K.B., Lee C.H., Kuo P.L. (2014). Substrate stiffness regulates filopodial activities in lung cancer cells. PLoS One.

[23] Jung M., Kim D., Mun J.Y. (2020). Direct visualization of actin filaments and actin-binding proteins in neuronal cells. Front. Cell Dev. Biol..

[24] Song X., Chen X., Yamaguchi H., Mouneimne G., Condeelis J.S., Eddy R.J. (2006). Initiation of cofilin activity in response to EGF is uncoupled from cofilin phosphorylation and dephosphorylation in carcinoma cells. J. Cell Sci..

[25] Iida K., Yahara I. (1999). Cooperation of two actin-binding proteins, cofilin and Aip1, in Saccharomyces cerevisiae. Genes Cells.

[26] Gehler S., Shaw A.E., Sarmiere P.D., Bamburg J.R., Letourneau P.C. (2004). Brain-derived neurotrophic factor regulation of retinal growth cone filopodial dynamics is mediated through actin depolymerizing factor/cofilin. J. Neurosci..

[27] Fass J., Gehler S., Sarmiere P., Letourneau P., Bamburg J.R. (2004). Regulating filopodial dynamics through actin-depolymerizing factor/cofilin. Anat. Sci. Int..

[28] Coste B., Xiao B., Santos J.S., Syeda R., Grandl J., Spencer K.S., Kim S.E., Schmidt M., Mathur J., Dubin A.E., Montal M., Patapoutian A. (2012). Piezo proteins are pore-forming subunits of mechanically activated channels. Nature.

[29] Dombroski J.A., Hope J.M., Sarna N.S., King M.R. (2021). Channeling the force: piezo1 mechanotransduction in cancer metastasis. Cells.

[30] Lin Y.C., Guo Y.R., Miyagi A., Levring J., MacKinnon R., Scheuring S. (2019). Force-induced conformational changes in PIEZO1. Nature.

[31] Wang Y., Shibasaki F., Mizuno K. (2005). Calcium signal-induced cofilin dephosphorylation is mediated by Slingshot via calcineurin. J. Biol. Chem..

[32] Sun Y., Leng P., Guo P., Gao H., Liu Y., Li C., Li Z., Zhang H. (2021). G protein coupled estrogen receptor attenuates mechanical stress-mediated apoptosis of chondrocyte in osteoarthritis via suppression of Piezo1. Mol. Med..

[33] Tse J.R., Engler A.J. (2010). Preparation of hydrogel substrates with tunable mechanical properties. Curr. Protoc. Cell Biol. Chapter..

[34] Klein E.A., Yin L., Kothapalli D., Castagnino P., Byfield F.J., Xu T., Levental I., Hawthorne E., Janmey P.A., Assoian R.K.J.C.B.C. (2009). Cell-cycle control by physiological matrix elasticity and in vivo tissue stiffening.

[35] Marshall J. (2011). Transwell((R)) invasion assays. Methods Mol. Biol..

[36] Jia X., Yang J., Song W., Li P., Wang X., Guan C., Yang L., Huang Y., Gong X., Liu M., Zheng L., Fan Y. (2013). Involvement of large conductance Ca(2+)-activated K (+) channel in laminar shear stress-induced inhibition of vascular smooth muscle cell proliferation. Pflügers Archiv.

[37] Jia X., Yang Q., Gao C., Chen X., Li Y., Su H., Zheng Y., Zhang S., Wang Z., Wang H., Jiang L.H., Sun Y., Fan Y. (2021). Stimulation of vascular smooth muscle cell proliferation by stiff matrix via the IK(Ca) channel-dependent Ca(2+) signaling. J. Cell. Physiol..

[38] Yang S., Miao X., Arnold S., Li B., Ly A.T., Wang H., Wang M., Guo X., Pathak M.M., Zhao W., Cox C.D., Shi Z. (2022). Membrane curvature governs the distribution of Piezo1 in live cells. Nat. Commun..

[39] Suzuki R., Inoh Y., Yokawa S., Furuno T., Hirashima N. (2021). Receptor dynamics regulates actin polymerization state through phosphorylation of cofilin in mast cells. Biochem. Biophys. Res. Commun..

[40] Zhou H., Wang J., Zhu P., Hu S., Ren J. (2018). Ripk3 regulates cardiac microvascular reperfusion injury: the role of IP3R-dependent calcium overload, XO-mediated oxidative stress and F-action/filopodia-based cellular migration. Cell. Signal..

[41] Holt J.R., Zeng W.Z., Evans E.L., Woo S.H., Ma S., Abuwarda H., Loud M., Patapoutian A., Pathak M.M. (2021). Correction: spatiotemporal dynamics of PIEZO1 localization controls keratinocyte migration during wound healing. eLife.

[42] Kim M.C., Silberberg Y.R., Abeyaratne R., Kamm R.D., Asada H.H. (2018). Computational modeling of three-dimensional ECM-rigidity sensing to guide directed cell migration. Proc. Natl. Acad. Sci. U. S. A..

[43] Yu S., Deng R., Wang W., Zou D., He L., Wei Z., Pan Y., Li X., Wu Y., Wang A., Chen W., Zhao Y., Lu Y. (2024). Pharmacological manipulation of TRPC5 by kaempferol attenuates metastasis of gastrointestinal cancer via inhibiting calcium involved in the formation of filopodia. Int. J. Biol. Sci..

[44] Jacquemet G., Baghirov H., Georgiadou M., Sihto H., Peuhu E., Cettour-Janet P., He T., Perala M., Kronqvist P., Joensuu H., Ivaska J. (2016). L-type calcium channels regulate filopodia stability and cancer cell invasion downstream of integrin signalling. Nat. Commun..

[45] Hotulainen P., Llano O., Smirnov S., Tanhuanpaa K., Faix J., Rivera C., Lappalainen P. (2009). Defining mechanisms of actin polymerization and depolymerization during dendritic spine morphogenesis. J. Cell Biol..

[46] Meng Y., Takahashi H., Meng J., Zhang Y., Lu G., Asrar S., Nakamura T., Jia Z. (2004). Regulation of ADF/cofilin phosphorylation and synaptic function by LIM-kinase. Neuropharmacology.

[47] Meng Y., Zhang Y., Tregoubov V., Janus C., Cruz L., Jackson M., Lu W.Y., MacDonald J.F., Wang J.Y., Falls D.L., Jia Z. (2002). Abnormal spine morphology and enhanced LTP in LIMK-1 knockout mice. Neuron.

[48] Ding Y., Milosavljevic T., Alahari S.K. (2008). Nischarin inhibits LIM kinase to regulate cofilin phosphorylation and cell invasion. Mol. Cell Biol..

[49] Kang C.G., Han H.J., Lee H.J., Kim S.H., Lee E.O. (2015). Rho-associated kinase signaling is required for osteopontin-induced cell invasion through inactivating cofilin in human non-small cell lung cancer cell lines. Bioorg. Med. Chem. Lett.

[50] Li X., Ma G., Guo W., Mu N., Wang Y., Liu X., Su L. (2021). Hhex inhibits cell migration via regulating RHOA/CDC42-CFL1 axis in human lung cancer cells. Cell Commun. Signal..

[51] Oleinik N.V., Krupenko N.I., Krupenko S.A. (2010). ALDH1L1 inhibits cell motility via dephosphorylation of cofilin by PP1 and PP2A. Oncogene.

[52] Morena F., Argentati C., Caponi S., Luchtefeld I., Emiliani C., Vassalli M., Martino S. (2024). Piezo1 - Serine/threonine-protein phosphatase 2A - Cofilin1 biochemical mechanotransduction axis controls F-actin dynamics and cell migration. Heliyon.

[53] Xiong Y., Dong L., Bai Y., Tang H., Li S., Luo D., Liu F., Bai J., Yang S., Song X. (2022). Piezo1 activation facilitates ovarian cancer metastasis via Hippo/YAP signaling axis. Channels.

[54] Ma M., Li J., Li X., Jing M., Wang L., Jiang Y., Yang Z., He J., Wang M., Liu H., Chen Y., Mi K., Wang L., Fan J., Du H. (2025). Piezo1 activation improves NSCLC liver metastasis immunotherapy by overriding matrix stiffness-mediated bimodal PD-L1/CXCL10 regulation. Adv. Sci. (Weinh.).

