# Supplementary material for: Stiff matrix promotes lung cancer cell migration through down-regulating the Piezo1 channel expression to facilitate Ca2+-dependent filopodia formation
Source: Mater Today Bio. 2026 Jan 12;37:102786. doi: 10.1016/j.mtbio.2026.102786 (PMC12828606; doi:10.1016/j.mtbio.2026.102786)
Supplement: Multimedia component 1 [file mmc1.docx]

**Supplementary data**


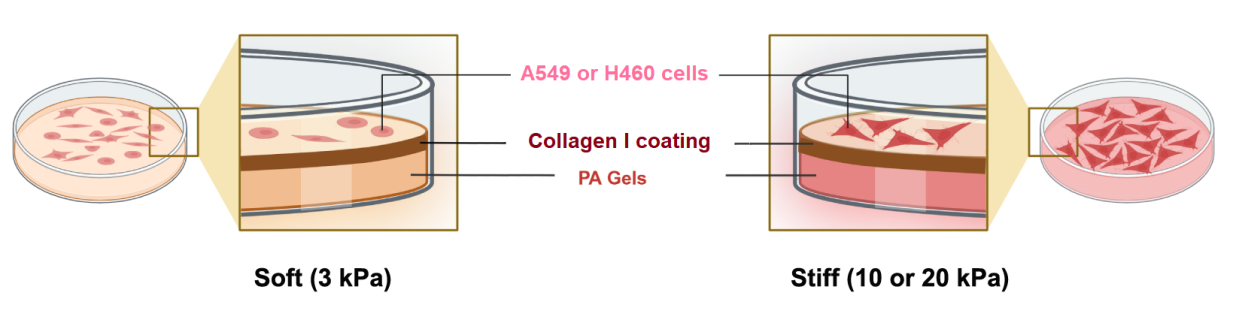


**Figure S1. Schematic diagram of A549 or H460 cells growing on soft (3kPa) and stiff (10 and 20 kPa) PA gels coated with collagen I.**


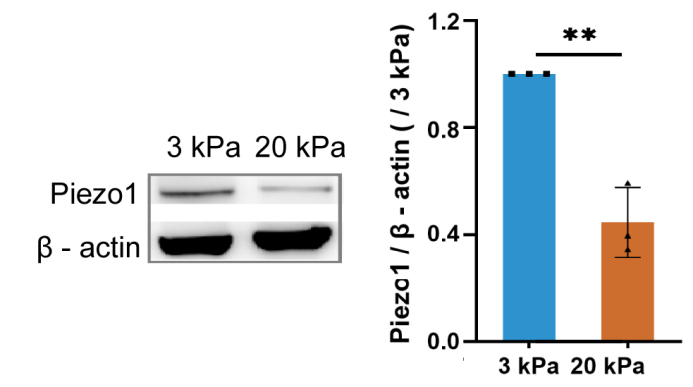


**Figure S2. Stiff substrate decreased the total Piezo1 protein expression.** Representative images of western blotting (left) and statistical analysis of data from three (right) independent experiments. All data were normalized to that of 3 kPa group. Data were presented as mean ± SD. ^**^*P* < 0.01.


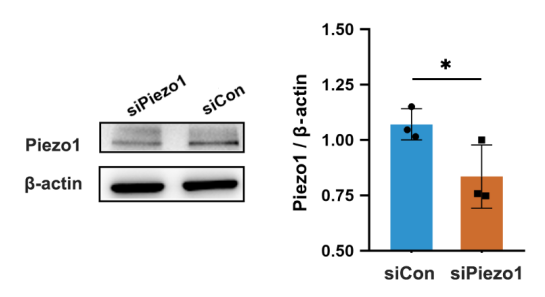


**Figure S3. Specific siRNA transfection significantly decreased the Piezo1 protein expression.** Data are presented as mean ± SD. n = 3, *P ≤ 0.5.


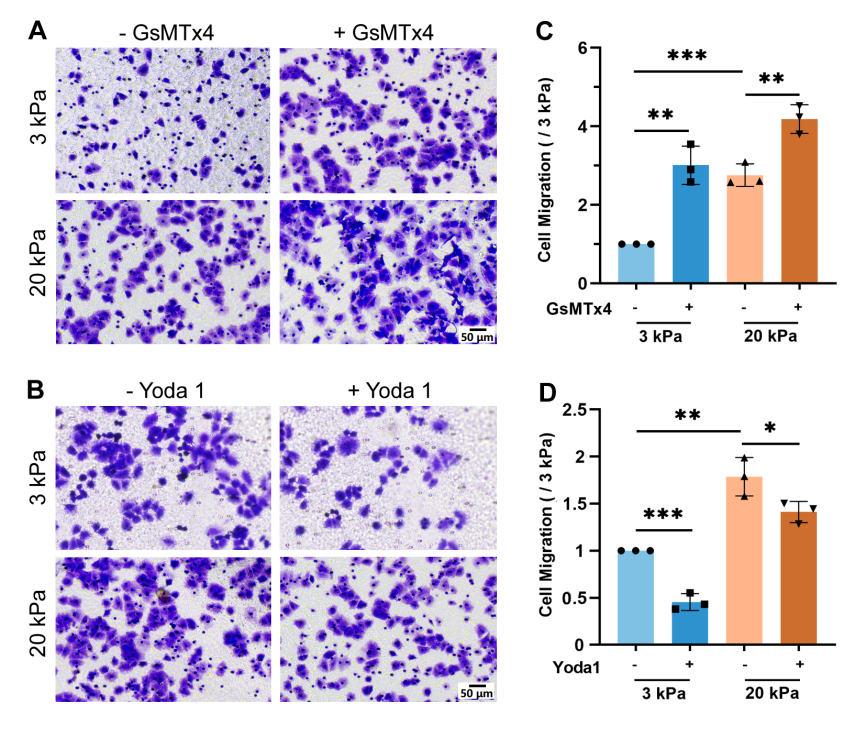


**Figure S4. Piezo1 channel negatively regulates substrate stiffness-induced H460 cell migration.** (A, C) Piezo1 channel blockade with GsMTx4 promotes cell migration on both soft and stiff substrates. (B, D) Piezo1 channel activation with Yoda1 inhibits cell migration on both soft and stiff substrates. Representative images of migrated cells stained with crystal violet (10x, A-B) and statistical analysis of data from three independent experiments (C-D). Scale bar: 50 μm. All data were normalized to the 3 kPa group. Data were presented as mean ± SD. *p < 0.05, **p < 0.01, ***p < 0.001.


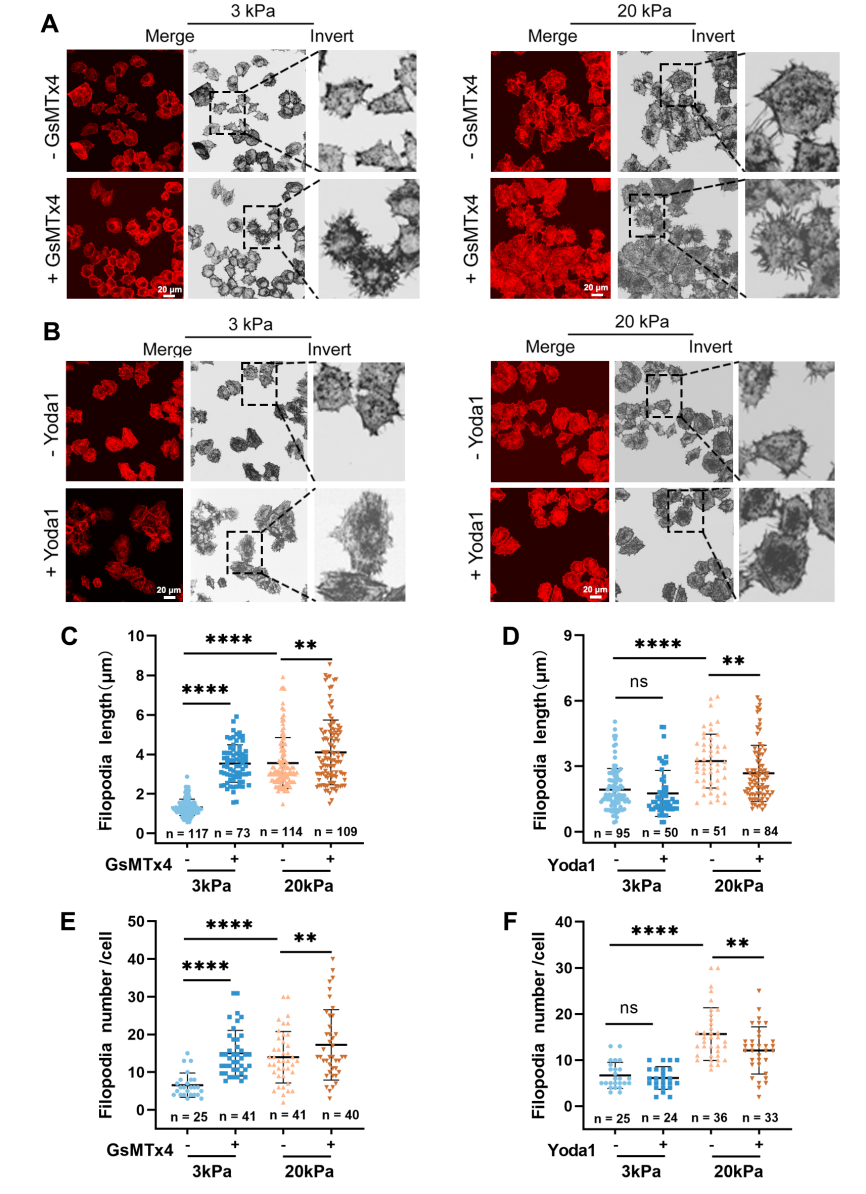


**Figure S5. Piezo1 channel negatively regulates stiff substrate-induced filopodia formation in H460 cells.** (A, C, E) Stiff substrate promotes filopodia formation in cells, and Piezo1 channel blockade with GsMTx4 further promotes filopodia formation in cells on both soft and stiff substrates. (B, D, F) Piezo1 channel activation with Yoda1 further inhibits filopodia formation in cells on the stiff substrates but has no effect in cells on the soft substrates. Representative images of filopodia morphology (A and B) and statistical analysis of the filopodia length (C and D) and number (E and F) from indicated number of cells. Red, F‐actin staining with rhodamine-labeled phalloidin; blue, nucleus staining with Hoechst 33342. All data were normalized to that of the 3 kPa group. Scale bar: 20 μm. Data were presented as mean ± SD. ^**^*P* < 0.01; ^****^*P* < 0.0001; ns, not significant.


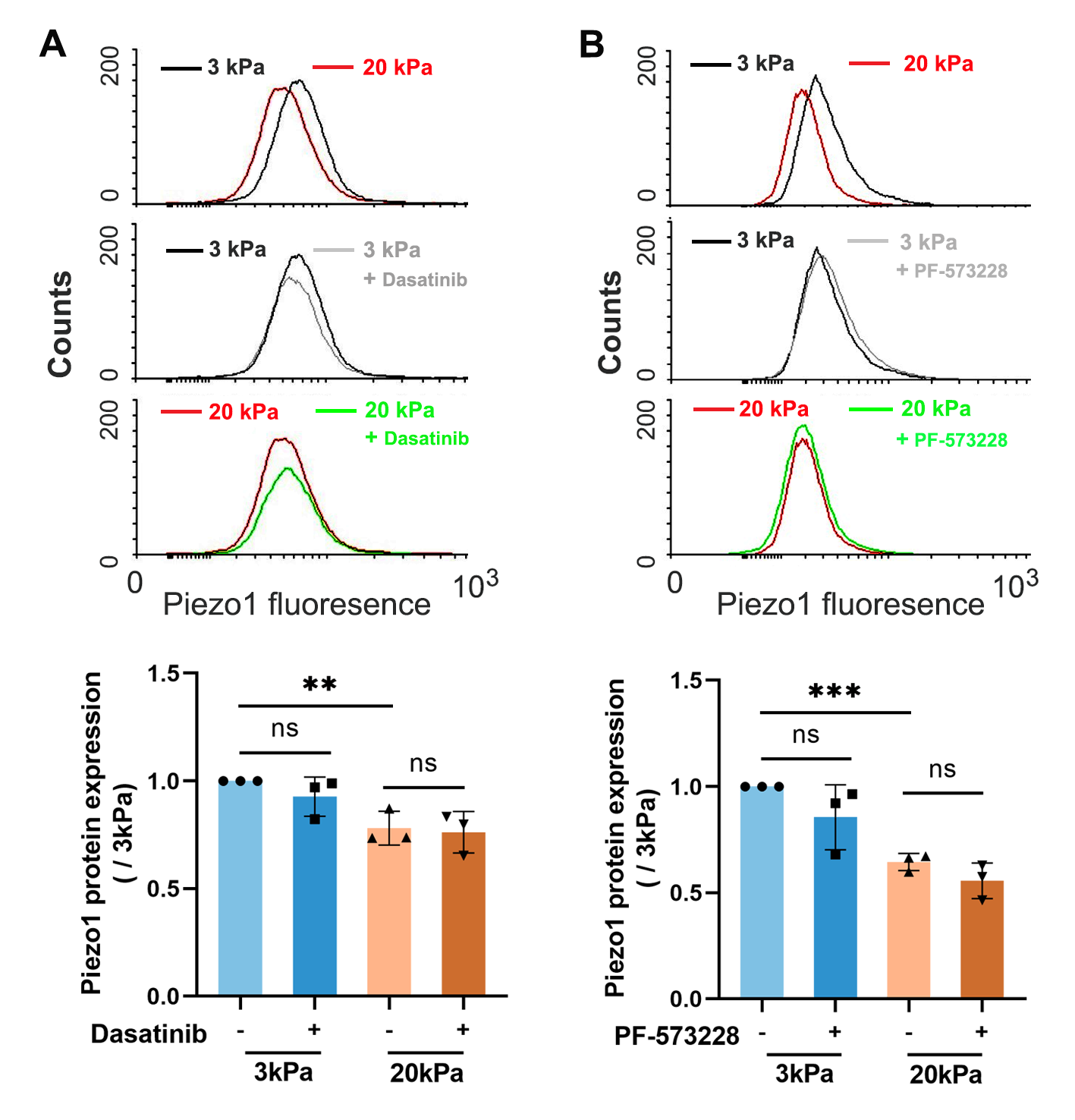


**Figure S6.** The Piezo1 channel expression of A549 cells on soft and stiff substrates with or without inhibition of YAP nuclear translocation with Dasatinib (A) or FAK activation with PF-573228 (B), respectively. Representative images of flow cytometry (upper) and data analysis from rom three independent experiments (bottom). All data were normalized to that of 3 kPa group. Data were presented as mean ± SD. ^**^*P* < 0.01; ^***^*P* < 0.001; ns, not significant.
